# Measles Immunity Status of Greek Population after the Outbreak in 2017–2018: Results from a Seroprevalence National Survey

**DOI:** 10.3390/vaccines11071220

**Published:** 2023-07-09

**Authors:** Asimina Nasika, Zacharoula Bogogiannidou, Varvara A. Mouchtouri, Katerina Dadouli, Maria A. Kyritsi, Alexandros Vontas, Ioanna Voulgaridi, Zafeiris Tsinaris, Konstantina Kola, Alexia Matziri, Athanasios G. Lianos, Fani Kalala, Efthimia Petinaki, Matthaios Speletas, Christos Hadjichristodoulou

**Affiliations:** 1 Laboratory of Hygiene and Epidemiology, Faculty of Medicine, University of Thessaly, 41222 Larissa, Greece; anasika@med.uth.gr (A.N.); zbogogiannidou@uth.gr (Z.B.); mouchtourib@med.uth.gr (V.A.M.); adadouli@uth.gr (K.D.); mkiritsi@med.uth.gr (M.A.K.); avontas@uth.gr (A.V.); ioavoulg@uth.gr (I.V.); ztsinaris@uth.gr (Z.T.); kokola@uth.gr (K.K.); alexmatz@uth.gr (A.M.); atlianos@uth.gr (A.G.L.); 2 Department of Immunology and Histocompatibility, Faculty of Medicine, University of Thessaly, 41500 Larissa, Greece; fkalala@uth.gr (F.K.); maspel@med.uth.gr (M.S.); 3 Department of Microbiology, University Hospital of Larissa, University of Thessaly, 41500 Larissa, Greece; petinaki@med.uth.gr

**Keywords:** measles, seroprevalence, immunity, anti-measles IgG antibodies, Greece

## Abstract

Accurate data on susceptibility rates against measles in the general population of Greece are scarce. Many studies have estimated the vaccination coverage, but none have calculated the nationwide immunity rate, including all age groups, against the measles virus. The purpose of our study was to determine the measles immunity status, especially after the latest outbreak in 2017–2018. In total, 3972 leftover blood samples were obtained during 2020–2021. They were collected from a nationwide laboratory network using a geographically stratified sampling strategy and were tested for the presence of measles-specific IgG antibodies. The overall crude seroprevalence was calculated to be 89.6% and the adjusted was 89.8% (95% CI: 88.8–90.8%). There was no statistically significant difference in seropositivity between sexes (*p* = 0.783). Higher immunity rates and antibody concentrations were found in older age groups ≥41 years old (94.9%, 95% CI: 93.7–95.9%, and 730.0 mIU/mL) in comparison with younger individuals aged 1–40 years old (83.4%, 95% CI: 81.6–85.7%, and 616.5 mIU/mL). Comparing the seroprevalence among the Nomenclature of Territorial Units for Statistics (NUTS 2), a statistically significant difference was estimated among them (<0.001). The two regions where higher measles incidence was observed during the 2017–2018 outbreak, Eastern Macedonia and Thrace, and Western Greece, were among the four regions with lower seropositivity (84.6%, 95% CI: 79.9–89.4%, and 85.9%, 95% CI: 81.4–90.4%, respectively). Our study showed a measles immunity gap that affects the younger age groups and makes a new measles outbreak likely. The enforcement of vaccination campaigns and addressing vaccine hesitancy could bridge it and achieve the required target of herd immunity.

## 1. Introduction

The adoption of childhood vaccinations during the second half of the 20th century has been one of the most successful and cost-effective public health policies [1,2]. The wide implementation of vaccination programs led to the control of several infectious diseases, as in the case of measles [3]. Measles is a highly contagious, serious disease caused by a virus belonging to the paramyxovirus family, and it is normally transmitted through direct contact and through the air [4]. It is documented that major epidemics occurred approximately every 2–3 years and measles caused an estimated 2.6 million deaths each year before the introduction of the measles vaccine and widespread vaccination [4].

In 1961, the vaccine was hailed as 100% effective and the first measles vaccine was licensed for public use in 1963 [5]. An improved version of the measles vaccine was created in 1968—a “weaker” version, where the virus was passed through chick embryo cells 40 times to weaken it—and in 1971, it was followed by a combined developed vaccine against measles, mumps, and rubella, known as the MMR vaccine [5]. In 2018, about 86% of the world’s children received one dose of a measles vaccine by their first birthday through routine health services—up from 72% in 2000—and during 2000–2018, measles vaccination prevented an estimated 23.2 million deaths [4].

As measles has such a high infectivity rate, the threshold for community protection is also very high, requiring at least 95% immunity among the population to prevent epidemics [6]. Despite the availability of a safe and cost-effective vaccine, an observed failure to maintain very high rates of immunization—a result of many causes—is resulting in measles outbreaks [7]. In 2018, 353,236 measles cases were reported to the World Health Organization (WHO) and there were more than 140,000 measles deaths globally, mostly among children under the age of five [4]. Global measles deaths continued to climb prior to the COVID-19 pandemic. In 2019, the highest number of reported cases in the last 23 years was recorded, at 873,022 cases, and there were over 207,000 measles deaths globally [5].

In Greece, around 1975, the measles vaccine started being administered to boys and girls aged 1 year, while it was introduced in the National Immunization Program as mandatory in 1981 [8,9]. Throughout the last two decades, the incidence of measles in Greece has presented a constant decline. However, sporadic clusters or even outbreaks are still recorded. The three last post-honeymoon outbreaks—the ‘honeymoon’ consists of the period following vaccine introduction, where cases dropped substantially—in Greece occurred in 2005–2006, 2010–2011, and the most extensive in 2017–2018 [10,11,12]. During the last epidemic in 2017–2018, 3259 cases were reported through the mandatory notification system in effect for measles, and 2104 (64.5%) of them were under the age of 14 years, including 833 children (25.5%) aged 1–4 years old [9,12,13]. The majority of cases concerned largely under-vaccinated groups of the Roma population (almost 60%), young adults aged 25–44 years old, and health workers with no or an incomplete history of vaccination [14]. The highest incidence was observed in the region of Western Greece and Eastern Macedonia—Thrace [9].

The European Centre for Disease Prevention and Control (ECDC) classifies Greece among the countries with >95% vaccination coverage with one dose of a measles-containing vaccine, while vaccination coverage with a second dose is estimated to be ≤89% [15,16,17]. Significant heterogeneity in vaccination coverage has been observed between different population samples. A recent study conducted in 2020–2021 estimated that MMR coverage among undergraduate Health Science students is about 96.1%, while a national survey conducted in 2017 found very low vaccination coverage for Roma children, based on vaccination documents; 47.9% of them were vaccinated with one MMR dose and only 7.6% with two doses [18,19].

A number of studies have been conducted in order to estimate the measles vaccine coverage, but only a few measured antibodies in serum samples in specific populations, and not at the national level [18,19,20,21,22]. The aim of our study was to calculate the Greek population’s immunity levels by measuring specific IgG anti-measles antibodies in serum samples representative of all Greek regions and age groups and assess whether seropositivity levels were associated with the incidence of measles in different regions.

## 2. Materials and Methods

### 2.1. Study Design and Participants

Blood samples were collected by using the leftover sampling methodology (residual sera from the general population) after the epidemic of 2017–2018 in Greece, during 2020–2021 [23]. We applied a geographically stratified sampling strategy based on regional units (Nomenclature of Territorial Units for Statistics (NUTS) level 3) to produce a representative sample, taking into consideration age (1–24, 25–54, 55–64, 65–79, and ≥80 years) and sex distribution. The required sample size was determined to be 246 blood samples from each of the 13 NUTS level 2 regions, using a margin of error of ±5%, a confidence level of 95%, an 80% expected frequency, and the estimated population (estimated population of Greece on 1 January 2020). The sample size was calculated using Epi info. We collected additional samples from the regions in Greece where the capital and the second-largest cities are located. However, the number of actual samples collected differed from the pre-determined number of samples above. Owing to this, post-stratification weights were applied based on each region’s population, as well as its sex and age distribution. This adjustment helped to account for any overrepresentation or underrepresentation of certain demographic groups in the sample.

The leftover blood samples were collected from a nationwide laboratory network, including both public hospital and private laboratories. The samples were derived from individuals who visited the laboratories for routine screening, check-ups, chronic disease follow-ups, or other medical reasons. Age, sex, residence, and the date of blood sampling were recorded for each sample.

### 2.2. Laboratory Analysis

Samples were collected and stored at −80 °C at the Laboratory of Hygiene and Epidemiology, Medical School in Larissa, Greece. The presence of anti-measles IgG antibodies was evaluated using a commercially available kit (SERION ELISA classic Masern/Measles Virus Ig, Institut Virion/Serion GmbH, Wurzburg, Germany) for the enzyme-linked immunosorbent assay (ELISA) according to the manufacturer’s recommendations. Absorbance was read at 405 nm and the IgG antibody concentration was calculated using a standard curve with standards included in the kit, based on the 2nd and 3rd WHO International Standards for Anti-Measles as referred to by the manufacturer. The threshold of 200 mIU/mL is not only defined by the manufacturer, but is also used as a generally accepted protective threshold [24,25]. Thus, the antibody concentrations were interpreted as negative (<150 mIU/mL), equivocal (150–200 mIU/mL), and positive (>200 mIU/mL).

### 2.3. Statistical Analysis

Initially, we determined an unweighted relative frequency of all positive participants’ characteristics (age, sex, and area of residence)—this is the crude seroprevalence. The weighted proportions of positive tests in the countrywide sample were based on the sex and age distribution of the Greek population according to the 2020 estimated population [26]. The 95% confidence interval (CI) for weighted data was estimated using the Clopper–Pearson exact binomial interval. The concentration of antibodies was described using the mean and standard deviation (StD). The 95% CI for the mean was estimated using a normal distribution. Chi-square (*χ*^2^), Mann–Whitney U, and Kruskal–Wallis tests were used to assess differences in seropositivity and concentrations of antibodies as appropriate. To compare the relative incidence of measles between regions, we used binary logistic regression models. These models were adjusted for age and sex. We calculated the adjusted seroprevalence of the four regions with the lowest seropositivity and we used it as the reference group. For all analyses, a 5% significance level was set. All statistical analyses were performed using the Statistical Package for the Social Sciences software version 29.0 (SPSS Inc., Chicago, IL, USA).

### 2.4. Ethical Statement

The current study was approved by the Ethics Committee of the Faculty of Medicine, University of Thessaly (No. 186). A written consent form was signed by all of the participants and the serum samples were anonymized and encrypted during the study.

## 3. Results

In total, 3972 samples were collected during 2020–2021 from all regions of Greece and analyzed. Data on sex, age, and place of residence were available for 94.4%, 97.4%, and 97.9% of the total sample, respectively. The accurate age was available for 1512 participants, with the following characteristics: min = 1 year, max = 99 years, mean = 43.7 years, and median = 43 years.

According to Table 1, the crude seroprevalence for the population was 89.6% and the adjusted seroprevalence was 89.8 (95%, CI 88.8–90.8%). For 1354 positive participants, the accurate age was available with the following characteristics: min = 1 year, max = 99 years, mean = 45.2 years, and median = 46 years. There was no statistically significant difference in seroprevalence between sexes (*p* = 0.783), between large urban areas and the rest of the country (*p* = 0.991), or between the islands and the mainland (*p* = 0.523). However, there was a difference in seroprevalence between different age groups (*p* < 0.001). The proportion of positive samples increased with age, as we observed the highest adjusted seroprevalence in the following age groups: 55–64 (95.6%, 95% CI: 93.7–97.4%), 65–79 (94.9%, 95% CI: 93–96.7%), and 80+ (95.4%, 95% CI: 92.3–98.6%). Additionally, we found the adjusted seroprevalence to be significantly higher for individuals over 41 years old (94.9%, 95%CI: 93.7–95.9%) compared with those aged under 40 (83.4%, 95% CI: 81.6–85.7%) (*p* < 0.001). 

Regarding antibody concentration, we also found a significant difference between sexes (*p* = 0.003). There was a statistically significant difference in the medians of antibody concentrations between the age groups (*p* < 0.001), with the lowest concentration of 632.1 mIU/mL being observed in individuals aged 25–54 and the highest concentration of 749.6 mIU/mL in those aged 65–79. The median concentration demonstrated no difference when we compared large urban areas with the rest of the country and the islands with the mainland.

**Figure 1:**
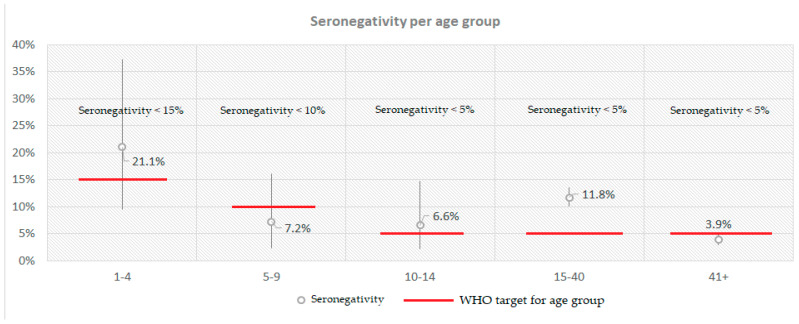
Calculated seronegativity for anti-measles IgG antibodies per age group compared with the respective WHO target in Greece in a post-measles-outbreak period.

Additionally, when comparing this study’s found seronegativity with the targets set for each age group by the WHO [27], children aged 1–4 (21.1%), 10–14 (6.6%) and individuals 15–40 years old (11.8%) did not meet them (Figure 1).

**Figure 2:**
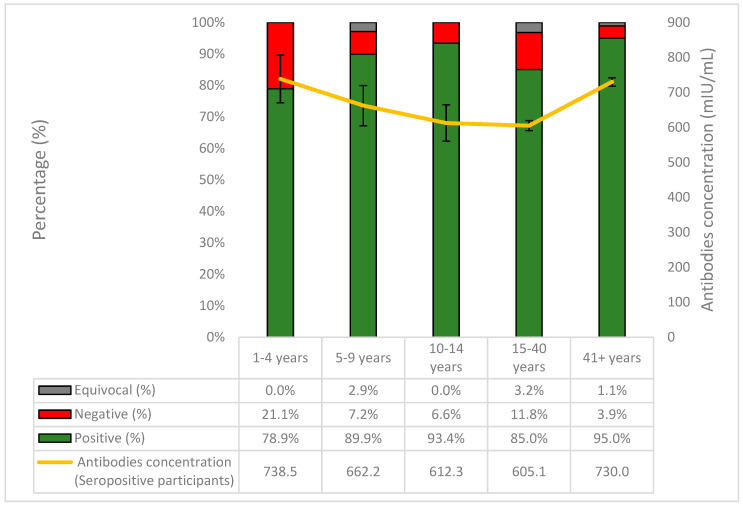
The percentages (%) of positive, negative, and equivocal samples of anti-measles IgG antibodies per age group and the course of the antibodies concentration in seropositive participants.

The highest proportion of seronegative samples was observed in those aged 1–4 years old (21.1%) and the lowest in individuals aged > 41 years old (3.9%) (Figure 2). The highest antibody concentrations, however, were found in those aged 1–4 years old (738.5 mIU/mL) and the lowest in those aged 15–40 years old (605.1 mIU/mL). The last age group also exhibited the highest proportion of equivocal samples (3.2%). Finally, seropositivity peaked in those aged >41 years old (95%), but was lowest in children aged 1–4 (78.9%).

According to Table 2 and Figure S1, when analyzing the presence of measles antibodies in different regions of Greece (NUTS 2), Eastern Macedonia and Thrace (84.6%, 95% CI: 79.9–89.4%) and Western Macedonia (85.2%, 95% CI: 78.1–92.3%) had the lowest seroprevalence. The lowest antibody concentrations were recorded in Eastern Macedonia and Thrace, at 610.7 mIU/mL, and Western Greece, at 636.9 mIU/mL. On the contrary, the highest seroprevalence was observed in the North Aegean Region (95.7%, 95% CI: 91.0–100%) and Peloponnese (95.6%, 95% CI: 92.6–98.7%). However, the highest antibody concentrations were found in South Aegean (730.9 mIU/mL) and in Central Macedonia (705.1 mIU/mL).

**Figure 3:**
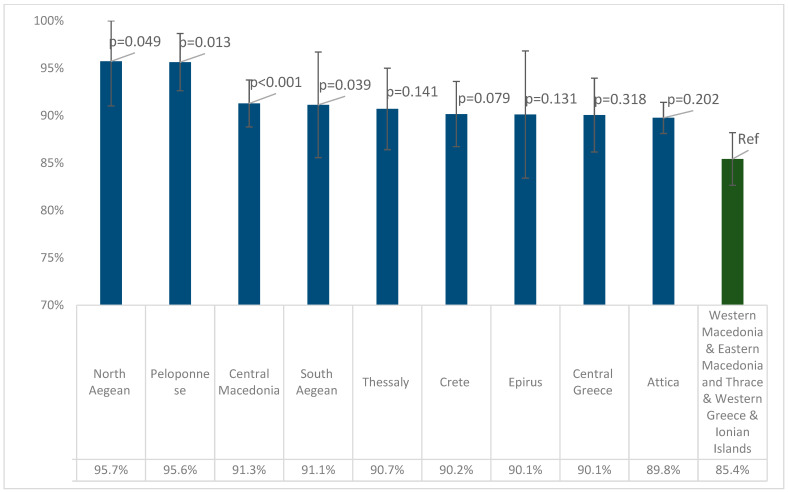
Pairwise comparisons of seropositivity for anti-measles IgG antibodies between the four regions with the lowest seropositivity and the remaining regions. *p*-values were derived from binary logistic regression models with IgG-positive (1/0) as the outcome variable, adjusted for age and sex. Error bars show 95% confidence intervals.

According to Table 2, the four regions with the lowest seropositivity were Western Macedonia, Eastern Macedonia and Thrace, Western Greece, and the Ionian Islands, and the seropositivity of these regions was 85.4% (95% CI 82.6–88.2%). Figure 3 depicts the pairwise comparisons of seropositivity between these four with the remaining regions. Peloponnese, Central Macedonia, and North and South Aegean had statistically significantly higher seropositivity than the four Regions with the lowest seroprevalence, while in Thessaly, Crete, Epirus, Central Greece, and Attica, higher seropositivity was calculated, but without a statistically significant difference.

## 4. Discussion

The aim of our study was to estimate the seroprevalence of measles in the general population of Greece. We found that almost 90% of individuals had serological proof of immunity against the measles virus in our study population, which indicates that about 10% were susceptible to measles. Similar seropositivity levels were calculated both in other Greek and European serosurveys; 89.3% of Greek multi-transfused patients were found to be positive, while in Germany, the seroprevalence in healthy participants was calculated to be 89.9% and that in Spain was 92.1% [22,28,29] Considering that at least 95% vaccination coverage with two doses of measles-containing vaccine is the widely accepted target for controlling the spread of the measles virus adopted by the WHO, there is an immunity gap against measles in the Greek population [6,30]. This specific target rate is important in order to protect infants, who are vulnerable to complications of measles and too young to receive the first dose of the vaccine [31]. This observed deviation from herd immunity allows the circulation and spread of the virus and makes the appearance of clusters or even outbreaks among susceptible individuals possible.

A typical example that depicts the danger posed by a low immunity level is the measles epidemic that took place in 2017–2018 as part of an outbreak in Europe [32]. In Greece, it began in May 2017 and continued until the end of 2018. A total of 968 measles cases were confirmed in 2017 and 2291 in 2018. The outbreak affected multiple regions in Greece, with the highest incidence reported in Western Greece and Eastern Macedonia and Thrace [9,13]. The seroprevalence in these regions was estimated in our study to be among the lowest in the country. This could be one explanation among others that answers the question of why the epidemic affected these specific regions more and, on the other hand, indicates that, despite the virus’ circulation, herd immunity could be hardly achieved through natural infection. It should be noted that two more regions were identified with similar levels of low seropositivity. Observing that, in the last 20 years, a post-honeymoon measles outbreak occurs every 5–7 years in Greece, we should be vigilant, especially in the areas with low seroprevalence, as 2023–2024 may be a year of the reintroduction and spread of the measles virus.

The last outbreak mainly occurred among the susceptible population, such as Roma (60.5%), children younger than 14 years old—even infants are included—and young adults aged 25–44 years old, unvaccinated or incompletely vaccinated healthcare workers, and people of foreign origin (10%). In 2017, a study performed on 251 Roma children calculated that only 42.9–47.9% of them had been vaccinated with at least one dose of MMR; 18.8–21% had received it by the age of 24 months old, while only 6.8–7.6% were recorded as being vaccinated with two doses [19]. This study indicates that Roma people are an under-vaccinated and vulnerable population and explains the high proportion of cases in this community.

The finding of a large number of seronegative people was reinforced in another study carried out before the epidemic in 2017–2018 [21]. They collected and tested 611 serum samples during June 2014–January 2016 from Northern Greece, with the age of the participants ranging from 10 days to 82 years old. Seroprevalence was found to be 82.07%. However, newborns and infants not eligible for vaccination were included, and this could explain the low level of seroprevalence that was found. If excluding 0–15-month-old babies, as the majority of them are under the vaccination age threshold according to our national vaccination schedule, seropositivity of almost 87.4% was calculated, further approaching our results. Either way, these levels of seropositivity are below those required for herd immunity and could explain the measles epidemic that followed. In our study, we excluded children under 1 year old.

However, even after the outbreak, the population’s immunity did not reach the desired level for measles elimination. A serosurvey calculating measles antibodies among multi-transfused patients with hemoglobinopathies conducted in Greece following the last measles outbreak showed that 89.3% of patients were found to be positive [22]. However, a group of frequently transfused patients does not appropriately represent the general population.

In another serosurvey performed in 2018–2019 on Greek soldiers aged 18.3 to 29.9 years old, the immunity rate against measles was calculated to be 80%, much lower than our result. It is also worth mentioning that, according to their vaccination certifications, 94.3% of the participants were fully vaccinated against measles and almost half of them in a timely manner [20]. The significant difference found between recorded immunization and observed seropositivity could bear clinical and epidemiological consequences and, therefore, highlights the importance of serosurveys. Based only on vaccination certification, it is possible to underestimate the risk of measles outbreaks.

According to a vaccination coverage study published in 2017, 97.3% of Greek preschool children aged 2–3 years were immunized with one dose of MMR [17]. However, a second dose is recommended for all children, which is essential to immunize the approximately 15% of them who do not develop protective immunity after their first dose [5]. Vaccination coverage of >95% was also found via the Child Vaccination Booklet in another published study conducted in the academic year 2020–2021 on health science undergraduate students aged 18–30 years old [18]. However, studies based on vaccination certifications of specific social groups may overestimate the immunity levels and provides the false impression of an ideal seroepidemiological status. As already mentioned before, the ECDC ranks Greece among the countries with vaccination coverage against measles of ≤89% in the general population [15]. 

We have to consider that the collection of our serum samples was conducted during a period overlapping the first years of the COVID-19 pandemic. An immunity gap had already been documented. There is an undeniable risk that the gap has now widened further, as it is commonly accepted that access to health facilities and, hence, vaccination—especially for minorities—was neglected during the pandemic. According to the WHO, in 2021, a record high of nearly 40 million children missed a measles vaccine dose: 25 million children missed their first dose and an additional 14.7 million children missed their second dose [33]. The suboptimal vaccination coverage, in conjunction with the lifting of public health measures, magnifies the threat of a measles outbreak with a higher number of cases, subsequent complications, and even deaths.

In our study, individuals younger than 40 years did not meet the WHO threshold to ensure herd immunity. This finding raises questions about the effectiveness of vaccination policy and enforcement, considering they were born after 1981, the year when vaccination against the virus became mandatory. This may be due to a variety of factors, such as: (1) unequal access to health facilities for minorities, (2) vaccine hesitancy, especially after the publication of the non-evidenced, incorrect causal association between the MMR vaccine and autism spectrum disorders, (3) primary vaccination failure due to failures in vaccine attenuation, vaccination regimens, or administration, and (4) secondary vaccination failure, with waning vaccine-induced immunity, meaning a decrease in circulating IgG antibodies some years after vaccination [34,35]. An inverse relationship has been documented; as the years pass after the second dose of measles vaccination, the proportion of vaccinated persons who become seronegative increases [36]. We should not forget that mothers may be included in the young adults group. Lower levels of maternal antibodies may pose a major problem for infants and lead to a gap in protection until the children can be vaccinated.

Another equally important reason for low seropositivity among younger age groups could be the delay in the administration of the second dose [36]. As mentioned earlier in a survey conducted on the Greek Air Force, even though the overwhelming majority of the recruits were fully vaccinated, half of them received the second dose of vaccine late [20]. Untimely vaccination has also been documented by another study performed in Greece’s second-largest city; a 21% delay rate for the MMR vaccine was recorded [37]. This delay may have a negative impact on induced immunity. An inverse correlation between antibody concentrations and the time elapsed between the two vaccinations has been proven [38]. The proportion of individuals with an equivocal or negative concentration was higher the longer the time period since the last vaccination [38].

As opposed to younger age groups, adults over 40 years old have reached the desired threshold set by the WHO. This finding could be explained by taking into consideration that these individuals had been naturally immunized against the virus, mainly during childhood. A significant number of serosurveys performed in Europe have arrived at similar conclusions, and it is supported that naturally acquired immunity can be preserved for a longer time period [36,39,40,41]. Besides longer-term humoral immunity, a higher antibody concentration was observed in the same age group, a result compatible with natural infection [39].

We found no statistically significant difference in seroprevalence against measles between females and males, and to our knowledge, sex differences regarding immunization coverage have not been suggested, with the exception of one study that showed significantly higher vaccination coverage in females [18].

Among seropositive individuals, as said before, a significant difference was observed between those younger than 40 years old and older individuals. This finding could also possibly demonstrate the most effective immunity through natural infection [39]. Comparing the levels of antibody concentrations among sexes, males seem to present higher concentrations, but this is not statistically significant and comes in contrast with the general acceptance that females typically develop higher immune responses to viral infections and vaccines [42]. However, comparing the concentrations among sexes per age group, males presented statistically significantly higher concentration in the younger age group “25–54”, a finding also supported by another study [43]. In any case, both sexes showed high antibody concentrations in all age groups, making them immune to measles.

Our study presents some limitations. The leftover sampling methodology could be considered one of them, as the non-random, convenient sampling may affect the representativeness of the samples collected. In nine regions, the required sample size was not collected. Furthermore, we did not have exact information about age, sex, or place of residence for a small number of samples. Thus, these samples were excluded during statistical adjustment. Finally, there was no possibility of distinguishing between vaccination-induced immunity and natural infection during laboratory analysis, and there was no access to vaccination records.

However, our study is superior on many other points. To our knowledge, this is the first national seroprevalence study on measles involving the general population, with all age groups and both sexes. The majority of studies have estimated the immunity level for measles in Greece based on vaccination records. However, vaccination coverage studies may not accurately reflect immunity on the grounds that they do not account for primary and secondary vaccination failure or the case of natural infection. Few serosurveys have been carried out in Greece and have focused only on specific groups, such as air force recruits or patients with hemoglobinopathies [20,22]. There has been only one serosurvey estimating measles immunity in every age group, but it was limited to northern Greece and was conducted in 2017, during the measles outbreak, without reflecting the possible impact of the outbreak on the immunity of the population [21].

## 5. Conclusions

Measles is a vaccine-preventable disease targeted for elimination in most WHO regions. Greece is considered by the European Regional Verification Commission for Measles and Rubella to have eliminated the endemic transmission of measles in 2020 [44]. Unfortunately, the seroprevalence we found was lower than the one required for herd immunity. As the virus has not been eliminated worldwide, the threat of its reintroduction and subsequent outbreak is imminent. With a view to increasing the immunity level, vaccination campaigns must be urgently launched, aiming to raise awareness about the benefits of vaccines and address the issues of vaccine hesitancy and misinformation. The enforcement of immunization activities among minorities with poor access to health services, such as Roma populations and asylum seekers, and better surveillance strategies should also be encouraged [44].

## Figures and Tables

**Table 1:** Crude, adjusted seroprevalence, and concentration of anti-measles IgG antibodies in Greece in a post-measles-outbreak period.

		Positive (N)	Negative (N)	Equivocal (N)	Total	Crude Seroprevalence	Adjusted Seroprevalence *	95% CI		Sig.**	Antibodies Concentration of Seropositive Participants (mIU/mL)	95% CI		Sig.
Overall		3558	327	87	3972	89.6%	89.8%	88.8%	90.8%	-	672.9	662.7	683.1	-
Sex	Male	1382	132	27	1541	89.7%	89.2%	87.6%	90.8%	0.783 ^C^	692.9	674.0	711.8	0.003 ^M-W^
	Female	1974	180	54	2208	89.4%	89.8%	88.5%	91.1%		656.9	645.9	667.9	
Age groups	1–24	810	110	35	955	84.8%	84.4%	81.9%	86.8%	<0.001 ^C^	640.5	609.4	671.6	<0.001 ^K-W^
	25–54	1420	160	39	1619	87.7%	87.6%	85.9%	89.3%		632.1	619.0	645.3	
	55–64	555	25	4	584	95.0%	95.6%	93.7%	97.4%		744.2	723.7	764.6	
	65–79	486	17	6	509	95.5%	94.9%	93.0%	96.7%		749.6	730.2	769.0	
	80+	168	6	2	176	95.5%	95.4%	92.3%	98.6%		722.4	687.3	757.4	
Age groups	1–40	1149	165	54	1368	84.0%	83.4%	81.6%	85.7%	<0.001 ^C^	616.5	593.4	639.6	<0.001 ^M-W^
	≥41	1513	62	18	1593	95.0%	94.9%	93.7%	95.9%		730.0	718.0	742.1	
Large urban areas (>500,000)		1532	152	27	1711	89.5%	90.3%	84.4%	94.1%	0.991 ^C^	681.3	663.7	698.8	0.163 ^M-W^
Rest of country		1949	170	58	2177	89.5%	89.0%	84.4%	92.1%		665.6	653.4	677.8	
Islands		487	39	11	537	90.7%	90.7%	80.5%	95.4%	0.523 ^C^	665.2	642.7	687.7	0.899 ^M-W^
Mainland		2994	283	74	3351	89.3%	89.2%	85.3%	92.4%		673.7	662.2	685.2	

* Adjusted seroprevalence was calculated using sera from participants for whom sex and age data were available; ** Comparison between positive and non-positive results. CI: Confidence Interval; C: Chi-Square test; M-W: Mann–Whitney U test; K-W: Kruskal–Wallis test.

**Table 2:** Crude and adjusted seroprevalence of anti-measles IgG antibodies and antibodies concentration per region in Greece during a post-measles-outbreak period.

Region (NUTS Level 2)	Positive (N)	Negative (N)	Equivocal (N)	Total	Crude Seroprevalence	Adjusted Seroprevalence	95% CI		Sig.**	Antibodies Concentration of Seropositive Participants (mIU/mL)	95% CI		Sig.
Attica	1188	132	10	1330	89.3%	89.8% *	88.1%	91.4%	<0.001 ^C^	672.8	651.5	694.0	<0.001 ^K-W^
Central Greece	204	19	4	227	89.9%	90.1%	86.2%	93.6%		693.1	658.5	727.7	
Thessaly	231	19	6	256	90.2%	90.7% *	86.4%	95.0%		642.9	608.4	677.4	
Western Greece	200	22	12	234	85.5%	85.9% *	81.4%	90.4%		636.9	605.3	668.5	
Epirus	97	4	5	106	91.5%	90.5% *	83.9%	97.0%		692.5	570.0	814.9	
Crete	261	23	5	289	90.3%	90.2%	86.7%	93.6%		641.5	611.4	671.6	
Central Macedonia	586	38	24	648	90.4%	91.3% *	88.8%	93.8%		705.1	685.2	725.1	
Ionian Islands	64	10	2	76	84.2%	85.8%	78.0%	93.6%		686.1	627.3	745.0	
North Aegean	68	3	1	72	94.4%	95.7% *	91.0%	100%		645.7	584.9	706.5	
South Aegean	94	3	3	100	94.0%	91.1%	85.6%	96.7%		730.9	676.9	784.8	
Peloponnese	220	9	0	229	96.1%	95.6% *	92.6%	98.7%		695.3	661.2	729.4	
Western Macedonia	80	14	3	97	82.5%	85.2%	78.1%	92.3%		652.5	602.8	702.2	
Eastern Macedonia and Thrace	189	27	10	226	83.6%	84.6% *	79.9%	89.4%		610.7	576.1	645.3	

* Adjusted seroprevalence was calculated using sera from participants for whom sex, age, and residence data were available; ** Comparison between positive and non-positive results. CI: Confidence Interval; C: Chi-Square test; K-W: Kruskal–Wallis test.

## Data Availability

The data are not publicly available as they contain sensitive information at the individual level.

## Supplementary Materials

The following supporting information can be downloaded at: https://www.mdpi.com/article/10.3390/vaccines11071220/s1, Figure S1: Adjusted seropositivity for anti-measles IgG antibodies per Region in Greece in a post measles outbreak period (NUTS level 2).
