# Supplementary material for: Measles Immunity Status of Greek Population after the Outbreak in 2017–2018: Results from a Seroprevalence National Survey
Source: Vaccines (Basel). 2023 Jul 9;11(7):1220. doi: 10.3390/vaccines11071220 (PMC10385097; doi:10.3390/vaccines11071220)
Supplement: Supplementary file 1 [file vaccines-11-01220-s001.zip › vaccines-2470550-supplementary.pdf]

Supplementary Material

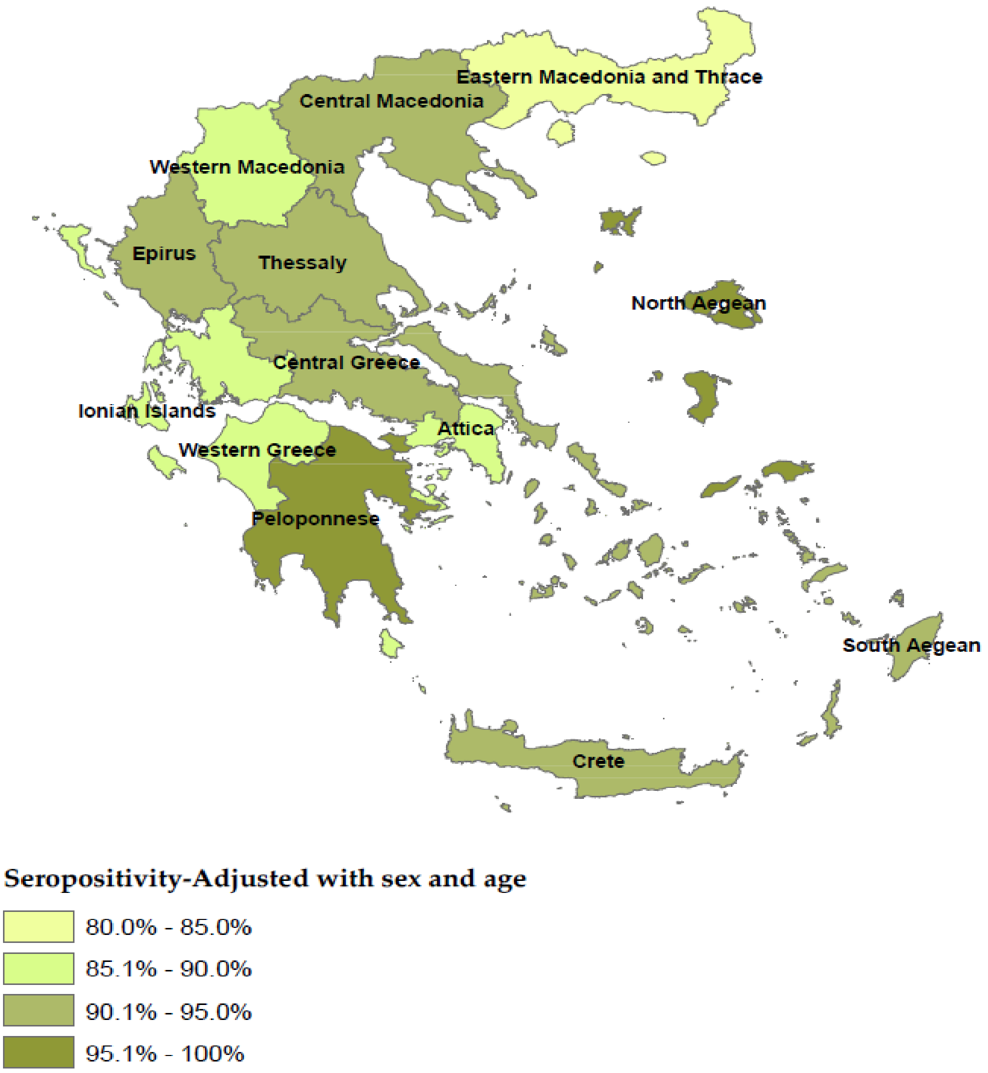

Figure S1. Adjusted seropositivity for anti-measles IgG antibodies per Region in Greece in a post measles outbreak period (NUTS level 2).
